# Supplementary material for: Fibroblast growth factor 2 inhibits myofibroblastic activation of valvular interstitial cells
Source: PLoS One. 2022 Jun 17;17(6):e0270227. doi: 10.1371/journal.pone.0270227 (PMC9205485; doi:10.1371/journal.pone.0270227)
Supplement: S1 Table — (DOCX) [file pone.0270227.s001.docx]

Supplementary table 1

Probes

| Gene ID | Details | Probe ID (Thermo Fisher) |
| --- | --- | --- |
| ACTA2 | Α smooth muscle actin | Hs00426835_g1 |
| BGLAP | Osteocalcin | Hs01587813_g1 |
| BMP2 | Bone morphogenic protein 2 | Hs00154192_m1 |
| BMP4 | Bone morphogenic protein 4 | Hs00370078_m1 |
| BMP6 | Bone morphogenic protein 6 | Hs00233470_m1 |
| CDH11 | Cadherin 11 | Hs00156438_m1 |
| COL1A1 | Collagen 1 α-chain 1 | Hs00164004_m1 |
| COL3A1 | Collagen 3 α-chain 1 | Hs00943809_m1 |
| ELN | Elastin | Hs00355783_m1 |
| FN1 | Fibronectin 1 | Hs00365052_m1 |
| ITGB1 | Integrin β 1 | Hs00559595_m1 |
| MMP1 | Matrix metalloprotease 1 | Hs00899658_m1 |
| MMP2 | Matrix metalloprotease 2 | Hs01548727_m1 |
| OPG | Osteoprotegerin | Hs00171068_m1 |
| OPN | Osteopontin | Hs00959010_m1 |
| RUNX2 | Runt-related transcription factor | Hs00231692_m1 |
| SCX | Scleraxis | Hs03054634_g1 |
| SMAD2 | SMAD2 | Hs00183425_m1 |
| SMAD3 | SMAD3 | Hs00969210_m1 |
| SP7 | Osterix | Hs00541721_m1 |
| TIMP1 | Tissue inhibitor of metalloprotease 1 | Hs00355335_g1 |
| VCL | Vinculin | Hs00419715_m1 |
| VIM | Vimentin | Hs00185584_m1 |

Supplemetary table 1 describes the panel of genes assayed in this study. Genes not described in the results section are detailed in the supplementary data
