# Supplementary material for: Fibroblast growth factor 2 inhibits myofibroblastic activation of valvular interstitial cells
Source: PLoS One. 2022 Jun 17;17(6):e0270227. doi: 10.1371/journal.pone.0270227 (PMC9205485; doi:10.1371/journal.pone.0270227)
Supplement: S2 Table — (DOCX) [file pone.0270227.s002.docx]

Supplementary table 2

Measures of RNA purity and integrity

| Measure | Details | Mean value | Range |
| --- | --- | --- | --- |
| A260/280 | Measure of DNA contamination | 2.11 | 2.07 – 2.16 |
| A260/230 | Measure of chemical carryover from RNA extraction | 1.48 | 0.38 – 2.39 |
| RIN | RNA integrity number | 9.97 | 9.9 – 10.0 |

Table 2 describes the quality control measures in our RNA extraction procedure
