# Supplementary figures and images for: Fibroblast growth factor 2 inhibits myofibroblastic activation of valvular interstitial cells
Source: PLoS One. 2022 Jun 17;17(6):e0270227. doi: 10.1371/journal.pone.0270227 (PMC9205485; doi:10.1371/journal.pone.0270227)

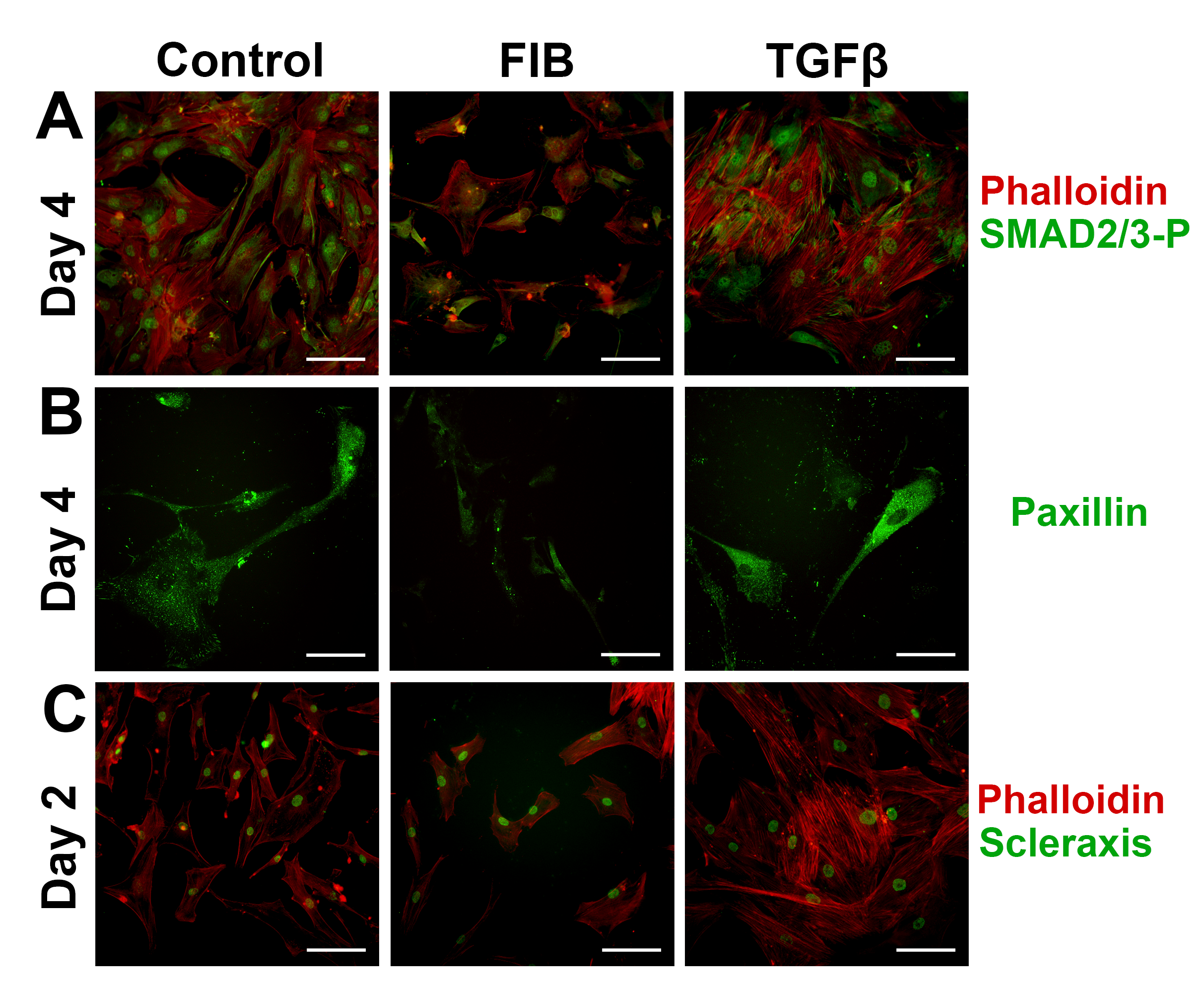

Supplement: S1 Fig — Immunocytochemistry from alternate timepoints (day 4 SMAD2/3, day 4 paxillin, and day 2 scleraxis) A) shows hVICs on day 4 stained for activated SMAD2/3 complex (green), counterstained with phalloidin (red), B) shows hVICs on day 4 stained for activated paxillin (green), C) shows hVICs on day 2 stained for activated scleraxis (green), counterstained with phalloidin (red). Scale bars represent 100μm. (TIF) [file pone.0270227.s003.tif]
